# Effectiveness of a Novel Qigong Meditative Movement Practice for Impaired Health in Flight Attendants Exposed to Second-Hand Cigarette Smoke

**DOI:** 10.3389/fnhum.2017.00067

**Published:** 2017-02-21

**Authors:** Peter Payne, Steven Fiering, James C. Leiter, David T. Zava, Mardi A. Crane-Godreau

**Affiliations:** ^1^Department of Microbiology and Immunology, Geisel School of Medicine at DartmouthLebanon, NH, USA; ^2^Department of Molecular and System Biology, Geisel School of Medicine at DartmouthLebanon, NH, USA; ^3^ZRT LaboratoryBeaverton, OR, USA

**Keywords:** second-hand cigarette smoke, flight attendants, Qigong, autonomic nervous system, somatics, resilience, stress in first responders, nicotine and fear learning

## Abstract

This single-arm non-randomized pilot study explores an intervention to improve the health of flight attendants (FA) exposed to second-hand cigarette smoke prior to the smoking ban on commercial airlines. This group exhibits an unusual pattern of long-term pulmonary dysfunction. We report on Phase I of a two-phase clinical trial; the second Phase will be a randomized controlled trial testing digital delivery of the intervention. Subjects were recruited in the Northeastern US; testing and intervention were administered in 4 major cities. The intervention involved 12 h of training in Meditative Movement practices. Based on recent research on the effects of nicotine on fear learning, and the influence of the autonomic nervous system on immune function, our hypothesis was that this training would improve autonomic function and thus benefit a range of health measures. Primary outcomes were the 6-min walk test and blood levels of C-reactive protein. Pulmonary, cardiovascular, autonomic, and affective measures were also taken. Fourteen participants completed the training and post-testing. There was a 53% decrease in high sensitivity C-Reactive Protein (*p* ≤ 0.05), a 7% reduction in systolic blood pressure (*p* ≤ 0.05), a 13% increase in the 6-min walk test (*p* ≤ 0.005), and significant positive changes in several other outcomes. These results tend to confirm the hypothesized benefits of MM training for this population, and indicate that autonomic function may be important in the etiology and treatment of their symptoms. No adverse effects were reported. This trial is registered at ClinicalTrials.gov (https://clinicaltrials.gov/ct2/show/NCT02612389/), and is supported by a grant from the Flight Attendant Medical Research Institute (FAMRI).

## Introduction

### Morbidities in flight attendants exposed to second-hand cigarette smoke

Flight attendants (FA) who flew before the ban on smoking in commercial aircraft (implemented progressively from 1988 to 2000) present with many of the co-morbidities of chronic obstructive pulmonary disease (COPD) but their pulmonary dysfunction differs from the standard definition of this disorder. At the time that they were hired, they were required to be in good health. They were exposed to second-hand cigarette smoke (SHCS) in the course of their often vigorous work activities, as well as to a wide range of other stressors (including interpersonal stress, aviation-associated threats and emergencies, disrupted diurnal rhythms and polluted air (Crawford and Holcomb, 1991; Grajewski et al., 2003; Repace, 2004; Sutton et al., 2007). Recent studies (Whelan et al., 2003; Arjomandi et al., 2009, 2012; McNeely et al., 2014) demonstrate significant rates of abnormal pulmonary function in this population: air trapping, reduced flow at mid-volume, reduced exercise tolerance, and significantly elevated rates of chronic bronchitis and sinusitis. There are also increased rates of cardiac disease, depression and anxiety (Pinkerton et al., 2012), sleep disturbances, skin and reproductive cancers and hearing loss (Czura, 2005; McNeely et al., 2014). Many of these symptoms are also co-morbidities of COPD (Shrikrishna and Hopkinson, 2012; Vestbo et al., 2013a).

The magnitude and nature of these pulmonary abnormalities do not meet the standard criteria of COPD using the Forced Expiratory Volume in one second (FEV1) to Forced Vital Capacity (FVC) ratio (Vestbo et al., 2013b) (now recognized as an unreliable criterion at the individual level). In addition, Han et al. (2010) have proposed that there is significant heterogeneity in the clinical symptoms of COPD and that a broader definition may be warranted to characterize its differing phenotypes. COPD is thought to be irreversible; and this and the hypothesized effects of cigarette smoke (CS)on the autonomic nervous system may in part account for the lasting nature of tobacco smoke exposure.

### Current lack of treatments aimed at FA

The nature of the morbidities experienced by FA exposed to SHCS is poorly defined. The significance and etiology of the clusters of pathology mentioned above is not clear. Apart from the screening studies noted above, little has been published that serves to define or to suggest treatments for these specific patterns of symptoms. Treatment tends to be directed at the primary symptom, and the possible significance of clusters of co-occurring symptoms is rarely examined.

Our work with FA has led us to examine the role of autonomic nervous system (ANS) activation in this population of first responders, whose job is “to perform vital crewmember functions onboard air carrier aircraft, including emergency functions for aircraft evacuations, firefighting, first aid, and response to security threats” (Ballough, 2004). In addition to its role as a principal cause of COPD (Moritsugu, 2007), cigarette smoke (CS) has been shown to affect the ANS via its influence on fear learning (Gould, 2006). Exposure to CS at the time of stressful experience potentiates fear conditioning (Davis et al., 2006; Cohen et al., 2007; Kutlu and Gould, 2015), and makes long-term post-traumatic stress and autonomic imbalance more likely (van der Velden et al., 2008). This imbalance of the ANS negatively affects the immune, cardiovascular, respiratory and musculoskeletal systems and may reinforce the pathological changes triggered directly in the lungs by CS. The possible role of autonomic disturbance induced by workplace exposure to SHCS in causing pulmonary, immune or affective disorders has largely been ignored, and as far as we know, no therapeutic approaches specifically aimed at such conditions have been proposed. Our selection of tests and of the MM/qigong intervention are informed by this perspective.

### Qigong

Qigong, a traditional Chinese health practice, has been used in China for hundreds of years in treating people with respiratory, autonomic and immune dysfunction (Johnson, 2002). Qigong includes a large range of practices using specific postures, movements, breathing patterns and visualizations to treat disease and improve health. Many of these practices have a broad positive influence on a range of pathologies (Lee, 2015; Dong and Bergren, 2016), and tend to restore physiological functions to normal range (Cohen, 1999). Qigong, Tai Chi and Hatha Yoga, have been proposed to constitute a novel category of exercise, “meditative movement” (MM) (Larkey et al., 2009). Specific MM practices have been shown to be an effective intervention for COPD (Ng et al., 2011; Liu et al., 2012; Chan et al., 2013) and to be equivalent or superior to conventional pulmonary rehabilitation in benefiting several of its symptoms (Xu et al., 2010; Ng et al., 2014). MM has also been shown to benefit the immune system, both reducing chronic systemic inflammation (Jahnke et al., 2010) and increasing the effectiveness of acquired immune response to infection (Wang et al., 2012). MM practices help restore functionality to the ANS (Sun and Yan, 1992; Lee et al., 2003a,b, 2004), and also benefit depression and anxiety (Payne and Crane-Godreau, 2013) and improve quality of life in many chronic diseases (Chan and Larson, 2015; Wang et al., 2015). MM also induces positive states of mind through focusing awareness on interoceptive and proprioceptive experiences (Payne and Crane-Godreau, 2013). We believe MM could prove to be of specific benefit to the particular problems experienced by FA exposed to SHCS, especially in view of the possible linking factor of autonomic imbalance.

### Aim

Our aim was to determine the effects of a novel form of MM/Qigong training by pre-and post-testing of pulmonary, autonomic and immune function in a cohort of FA previously exposed to occupational SHCS.

## Methods

All aspects of this study were approved by the Dartmouth IRB (CPHS # 28572). Additionally, this study is registered at ClinicalTrials.gov: (https://clinicaltrials.gov/ct2/show/NCT02612389/), and an earlier paper describing the proposed protocol has been published (Payne et al., 2016). All participants gave written informed consent using IRB-approved informed consent forms (Payne and Crane-Godreau, 2016a) prior to enrollment in the study. To preserve anonymity, each participant was assigned a code number, and the list of correspondences was kept in a locked drawer at the research office.

### Participants

#### Recruitment

Flight attendants (FA) were recruited from the Northeastern region of the US, in particular from areas where travel to the testing and training centers in Burlington VT, Woburn MA and New York NY was possible. Primary outreach was through existing flight attendant organizations and networks, social media and flight attendant events, as well as in collaboration with the Harvard School of Public Health Flight Attendant Health Study. Social media and audio-visual support were provided by the Department of Health Behavior at the Roswell Park Cancer Institute in Buffalo, NY.

#### Randomization and controls

This was a pilot study designed, in part, to select the most useful exercises, and a control group was not warranted. There was a baseline set of measurements for each subject before any MM intervention, and each participant acted as his/her own control so that data collected before the intervention could be compared to data collected after the MM training.

#### Inclusion and exclusion criteria

All participants were required to be non-smoking former or current (FA), who, in the course of their occupation, had been exposed to SHCS for at least 5 years. In addition, participants were required to attend at least 12 h of in-person training sessions and focus group feedback sessions provided in central locations over a 4-month period. They were also required to have devices on which to listen to audio and watch video instruction, and be willing to do so. Pregnancy or planned pregnancy, as well as cognitive impairment, severe emotional problems, or physical inability to perform the exercises, were grounds for exclusion. Participants were asked not to modify their lifestyle significantly during the study period apart from the practice required by the study. Since each participant acted as his/her own control, we did not exclude on the basis of medication use.

### Intervention

#### Experimental design

**Figure 1:**
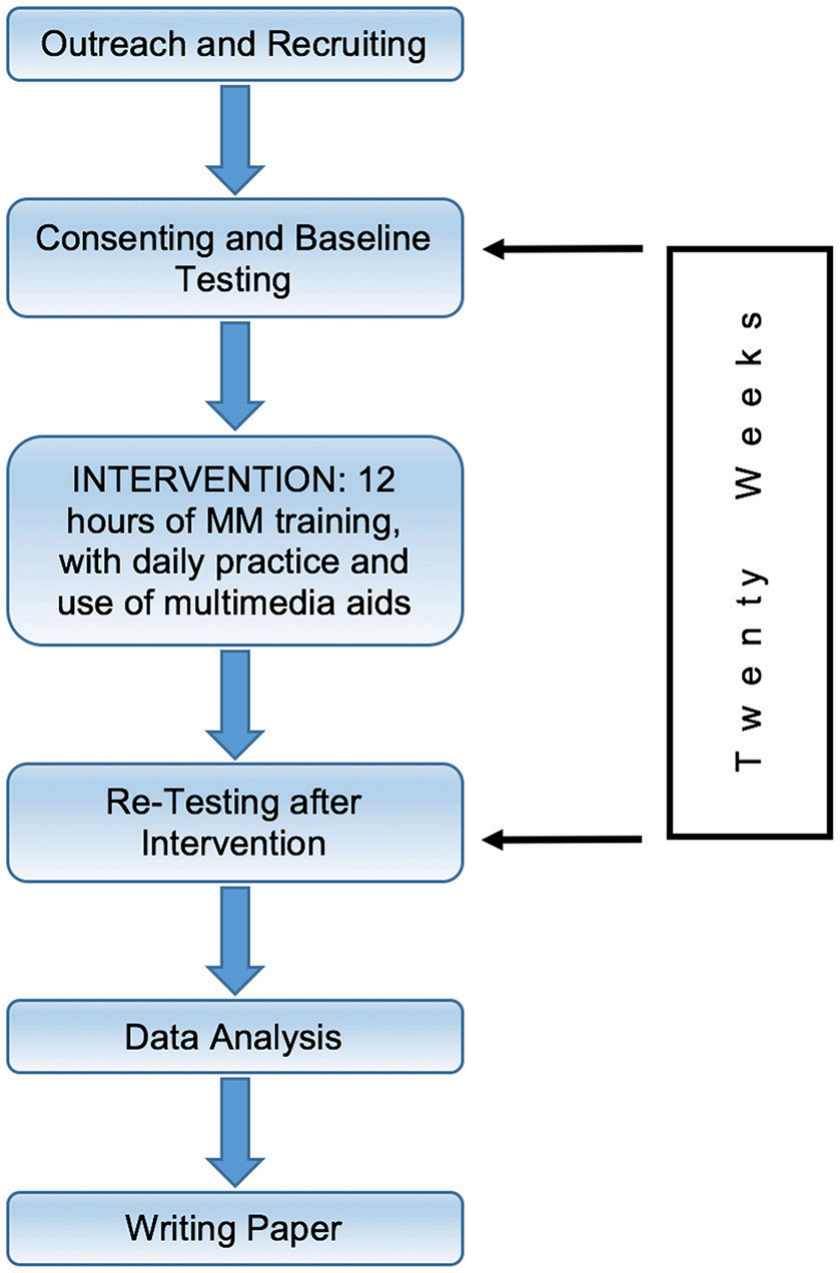
**Timeline Chart**. Timeline structure of study.

Figure 1 shows the overall experimental design of this study. Initial recruiting outreach was followed by enrolling and consenting participants, and administering pre-intervention testing. Next, participants received at least 12 h of face-to-face classroom instruction in selected qigong exercises. This stage was completed in 20 weeks for the majority of participants. Following the training, the same battery of tests was administered again.

#### Implementation of intervention

**Figure 2:**
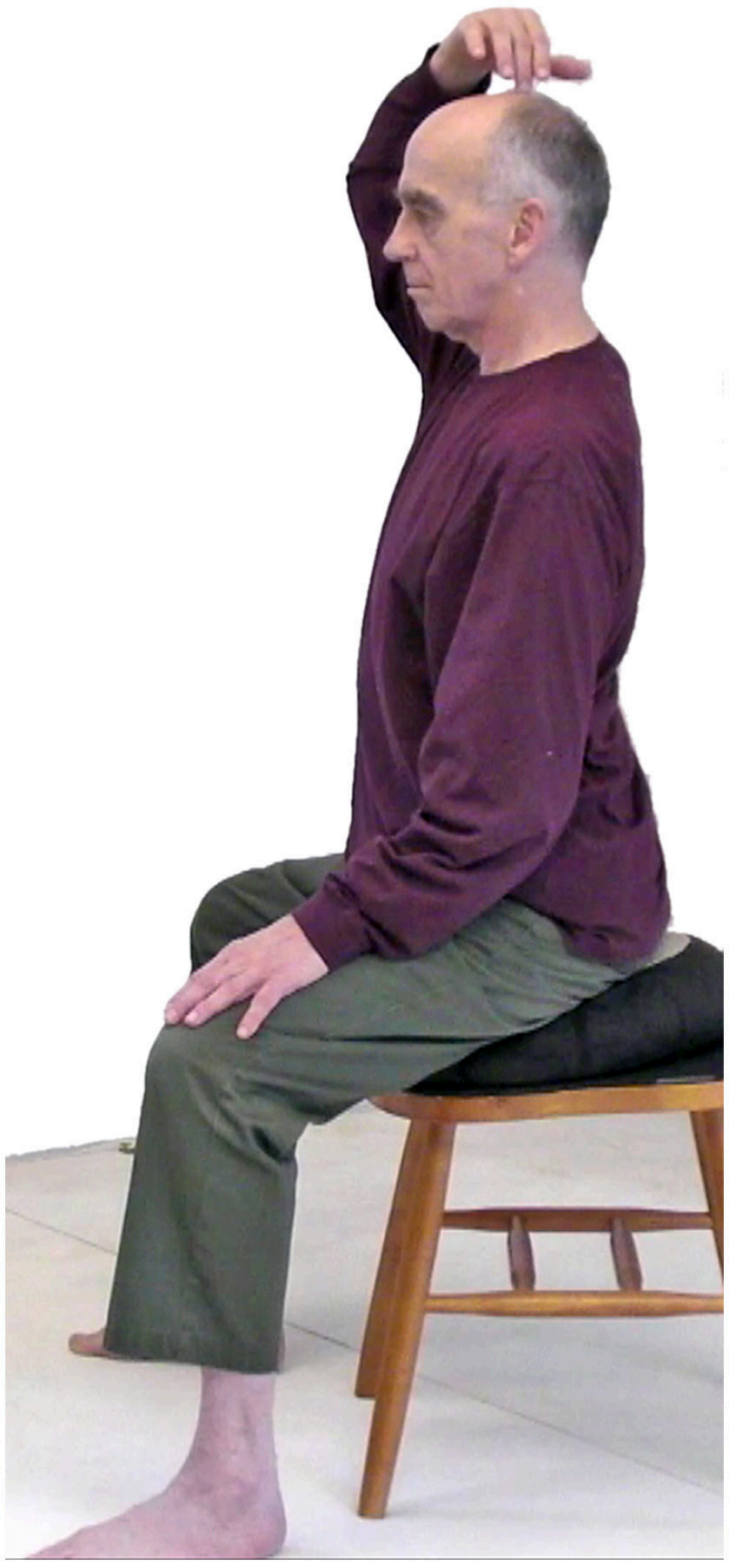
**Example of intervention practice**. One of the authors (PP) demonstrates sitting in balanced alignment, one of the practices taught in the MM intervention.

Training was commenced in three locations on three different dates: Burlington VT, New York NY, and Woburn MA. The content of the training shifted over time in response to participant compliance and feedback. All participants were asked to complete 12 mandatory hours of class time over a period of 4–5 months. Classes were offered at approximately 1 month intervals at convenient locations. There were between 2 and 7 participants at each class; at a given location classes were usually offered on two consecutive days to accommodate participants' schedules. There was an initial focus on exercises to be practiced at specific times, but in response to participant feedback the emphasis shifted to practices that could be integrated with daily life, such as standing, walking, and sitting. The practices involved static postures or slow and gentle movement, with encouragement to attend to kinesthetic and interoceptive experience and to focus on specific mental images. Figure 2 shows one of the authors (PP) demonstrating a practice. Detailed written descriptions, audio files, and access to videos of the exercises are available (Payne and Crane-Godreau, 2016b,c,d). A paper detailing the exercise selection process will be published. The instructors were well versed in the practice and instruction of qigong.

During class time participants were encouraged to provide feedback on their experience and practice of the exercises; all feedback was recorded in writing at the time. Participants were also asked to maintain a log of their experiences during the training. They were provided with printed sheets for recording their practice sessions as well as an online Survey Monkey page (Payne and Crane-Godreau, 2016e). All feedback was regularly reviewed by the researchers and instructors to evaluate the exercises for acceptability and compliance. The researchers communicated by email and phone with participants who were not responsive to requests for online feedback.

As described elsewhere (Payne et al., 2016), during the study we selected and refined the specific set of practices taught to participants, including simple postural practices, such as standing and sitting, moving practices, such as walking, and specific limb movements, as well as simple breathing. These practices were accompanied by directed attention to environmental, interoceptive and kinesthetic awareness. Participants were asked to integrate these practices into the course of their daily lives.

Participants were instructed to discontinue the exercises (in the class or at home) immediately if they experienced any of the following: dizziness, rapid or irregular heart-beat, sudden or excessive dyspnea, chest pain, significant pain anywhere in the body, pressure in the head, headache. During the classes the instructor carefully monitored the participants for signs of distress including facial paleness or redness, rapid breathing, sweating, or jerky movement.

### Outcome measures

Outcome measures were selected to monitor a wide range of health measures. Further technical details about these measures are available in a previously published protocol paper (Payne et al., 2016).

#### Primary outcome measures

- The 6 min walk test (6MWT), was conducted on an indoor level surface free of obstructions according to the guidelines established by the American Thoracic Society (ATS). Research assistants were on hand to assist participants with any difficulties. Maximum distances walked in 6 min were recorded (Hamilton and Haennel, 2000; Carter et al., 2003; Chandra et al., 2012; Chen et al., 2012).
- High sensitivity C-reactive protein (hs-CRP), a marker for systemic inflammation (Karadag et al., 2008), was measured from a blood sample obtained by finger-stick blood draw collected onto a prepared blood card. Samples were refrigerated, stored and then shipped to ZRT Laboratory where they were analyzed by mass spectrometry.

#### Secondary outcome measures

- Blood pressure was recorded in a seated position from the dominant arm, after the participant had been at the testing center for over 30 min. At least two readings were taken, 2 min apart, and if the readings differed by more than 10 points, a third reading was taken and the average value obtained and recorded.
- The COPD Assessment Test (CAT) (GlaxoSmithKline^1^; Feliz-Rodriguez et al., 2013) is a short questionnaire used to evaluate the perceived impact of respiratory dysfunction on an individual.
- Spirometry. FEV1, FVC, and Flow/Volume Curves were obtained according to the ATS guidelines (Society, 2002). We used specific validated spirometric cut points (Rabe et al., 2007). We recorded standard measures including FEV1, FVC, forced expiratory flow between 25 and 75% of capacity (FEF 25–75), peak expiratory flow (PEF) and Flow/Volume Curves, using the EasyOne Plus Frontline spirometry system.
- COMPASS 31: Each subject completed the COMPASS 31, a questionnaire for detecting symptoms of autonomic dysfunction (Sletten, 2012).
- Zung Self-Rating Anxiety and Depression Scales: These questionnaires, which provide self-report of symptoms of anxiety or depression, were completed by each subject in the presence of a research team member.
- Multidimensional Assessment of Interoceptive Awareness (MAIA) questionnaire (Mehling et al., 2012) evaluates the degree of awareness of interoceptive cues, and the degree of comfort with these cues.
- Blood vitamin D levels were determined from mass spectrometry analysis by ZRT Laboratory, from blood collected onto an absorbent paper blood card. The blood sample was obtained by finger-stick blood draw method. Blood and urine analysis was provided by ZRT Labs, Beaverton, Oregon.

### Statistical methods

We determined the required sample size for statistical significance based on a predicted difference of 46 meters in the 6MWT and an assessment of previous similar studies. Using this criterion, 20 participants in each group were needed to achieve a statistical power of 80% at a significance level of 5%. To compensate for anticipated drop-out, we recruited a total of 26 participants into the Selection group. Descriptive statistics, including mean, percent change and *p*-values, were used to describe and summarize baseline data. Handling missing data: In cases where data for a subject on one outcome measure is missing, we have chosen to eliminate that subject from the calculations for that measure. Significance levels for changes in measures from pre- to post-intervention test were determined using a one-tailed *t*-test. Comparisons between completers and non-completers were handled using a 2-tailed *t*-test. Categorical changes between these two groups were evaluated using a Chi-squared contingency table. Raw data and statistical calculations made are available (Payne and Crane-Godreau, 2016f).

## Results

### Participants

#### Participant flow

**Figure 3:**
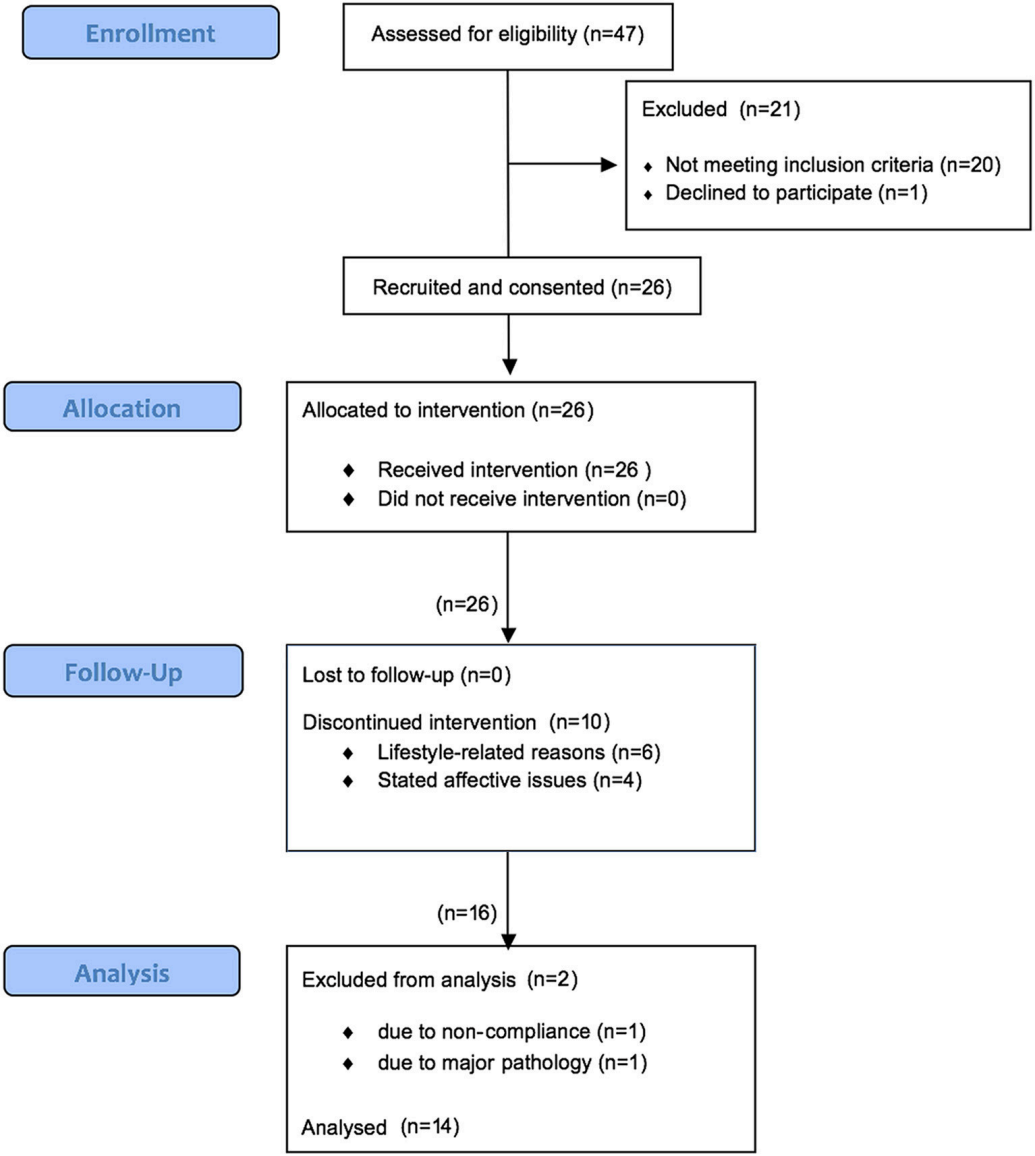
**Participant flow diagram**. 47 potential participants were approached, 26 were recruited. 10 discontinued the study and 2 were disqualified. 14 completed the study.

Figure 3 shows numbers of participants recruited and consented, reasons for non-completion, and numbers completing the study. Forty-seven (FA) were approached, and of those 20 did not meet inclusion criteria and one who met the criteria declined to participate. Twenty-six participants were then recruited and consented. All began receiving the MM training intervention. Prior to the completion of the post-intervention testing, 6 participants discontinued the study due to stated lifestyle conflicts and 4 discontinued due to stated affective issues. At the Analysis stage, 2 were disqualified by the researchers due to non-compliance and the disclosure of major pre-existing pathology. Fourteen completed the study and were analyzed.

#### Baseline demographic and clinical characteristics of each group

Table 1 shows the characteristics of the initial group of 26 recruits, and those of the group of 14 who completed the study. We made every effort to reach out to the widest range of this population, but 100% of those consented for the study were female. Two males contacted us about the study, but neither made an appointment for testing. Thirteen percent of our recruits were non-White. This is concordant with McNeely (2016) finding that nationwide only 11.5% of former or current FA between the ages of 50 and 90 years of age are non-White. Similarly, 87% of FA nationwide in the 50–90 age range are female (McNeely, 2016, p. 20017). These data may help to explain the low levels of diversity within this study population.

**Table 1:** **Baseline Participant Characteristics**.

	**All recruits *N* = 26**	**Completing group *N* = 14**
Total	26	14
Female	100%	100%
Average age	67.3	67.7
Age range	49–78	49–74
Number still employed as FA	2	1
Body mass index average	24.9	22.7
BMI range	17–37.2	17–36.3
White	23	14
African-American	2	0
Hispanic	1	0

*Baseline characteristics of all recruits and of those who completed the study. 26 were recruited, and 14 completed the study. All recruits were female, and all who completed the study were White*.

#### Adverse events

Meditative movement (MM) has few reported side effects (Ng and Tsang, 2009a), and no adverse events were reported by any of the participants.

### Outcome measures

#### Detailed results

Results are shown in Table 2. Raw data on which the results are based is available (Payne and Crane-Godreau, 2016f). We compared pre- and post-testing measures in two ways. First, we compared average values using Students *t*-test; and where appropriate, we also compared the number of people falling outside normal clinical range (in blood pressure measures, hs-CRP, and CATest) using a chi-squared test to determine the significance of the change from pre- to post-testing.

**Table 2:** **Outcome measures**.

**Outcome measure**		**Pre-test**	**Post-test**	**% change**	***p*-value**
Primary	6MWT	522 m	590 m	13%	≤0.004^**^
	hs-CRP	2.8 mg/l	1.3 mg/l	−53%	≤0.05^*^
Cardiovascular	Avg Systolic BP	130.5 mmHg	121.4 mmHg	−7%	≤0.05^*^
	Systolic BP %>140	43%	7%	36%	≤0.05^*^
	Avg Diastolic BP	81.4 mmHg	78.2 mmHg	−4%	ns
	Diastolic BP %>90	29%	7%	22%	ns
	Avg Pulse pressure	49.1 mmHg	43.2 mmHg	−12%	≤0.06^+^
	Resting HR	73.2	68.6	−6%	≤0.05^*^
	% change HR	14.4%	20.4%	42%	ns
	Avg Pulse pressure	49.1 mmHg	43.2 mmHg	−12%	≤0.06^+^
Pulmonary	CATest score	7.5	6.6	−12%	ns^+^
	FEV1	86.57	86.07	0%	ns
	FVC	90	86	−1%	ns
	25/75	79	73	−7%	ns
	PEF	92.7	95.9	3%	ns^+^
Autonomic	Compass 31	15	11.5	−26%	≤0.05^*^
Humoral	Vit D level	40.6	40.7	0%	ns
Interoceptive	MAIA	25.1	27.8	10%	≤0.05^*^
Affective	Zung Anxiety	29.4	26.9	−8%	≤0.05^*^
	Zung Depression	33.5	34	1%	ns

** , ≤ 0.01 significance level;

* , ≤ 0.05 significance level;

+ *, trending; ns: not significant. Baseline values, post training values, percent change, and significance levels*.

There was a highly significant improvement in the 6MWT comparing pre- to post-intervention distances, as well as a significant reduction in hs-CRP levels, systolic blood pressure and resting heart rate, as well as in the Zung Self-rating Anxiety Scale and the COMPASS 31 (indicating improved autonomic function). There was also a significant increase in the MAIA score, indicating improved interoceptive awareness. Non-significant trends were observed of decreased pulse pressure, reduced diastolic blood pressure, reduced blood oxygen saturation, reduced (improved) COPD Assessment Test score, and increased peak expiratory flow (PEF), as well as a substantial, although not significant, increase in the percent change in heart rate after the 6MWT. We note that there was a 22% reduction in the number of participants with a diastolic pressure above the clinical threshold of 90 mmHg and a 36% reduction in the number with a systolic pressure above 140, although only the figure for systolic blood pressure reached significance. Vitamin D levels did not change.

#### Graphical summary of key results

**Figure 4:**
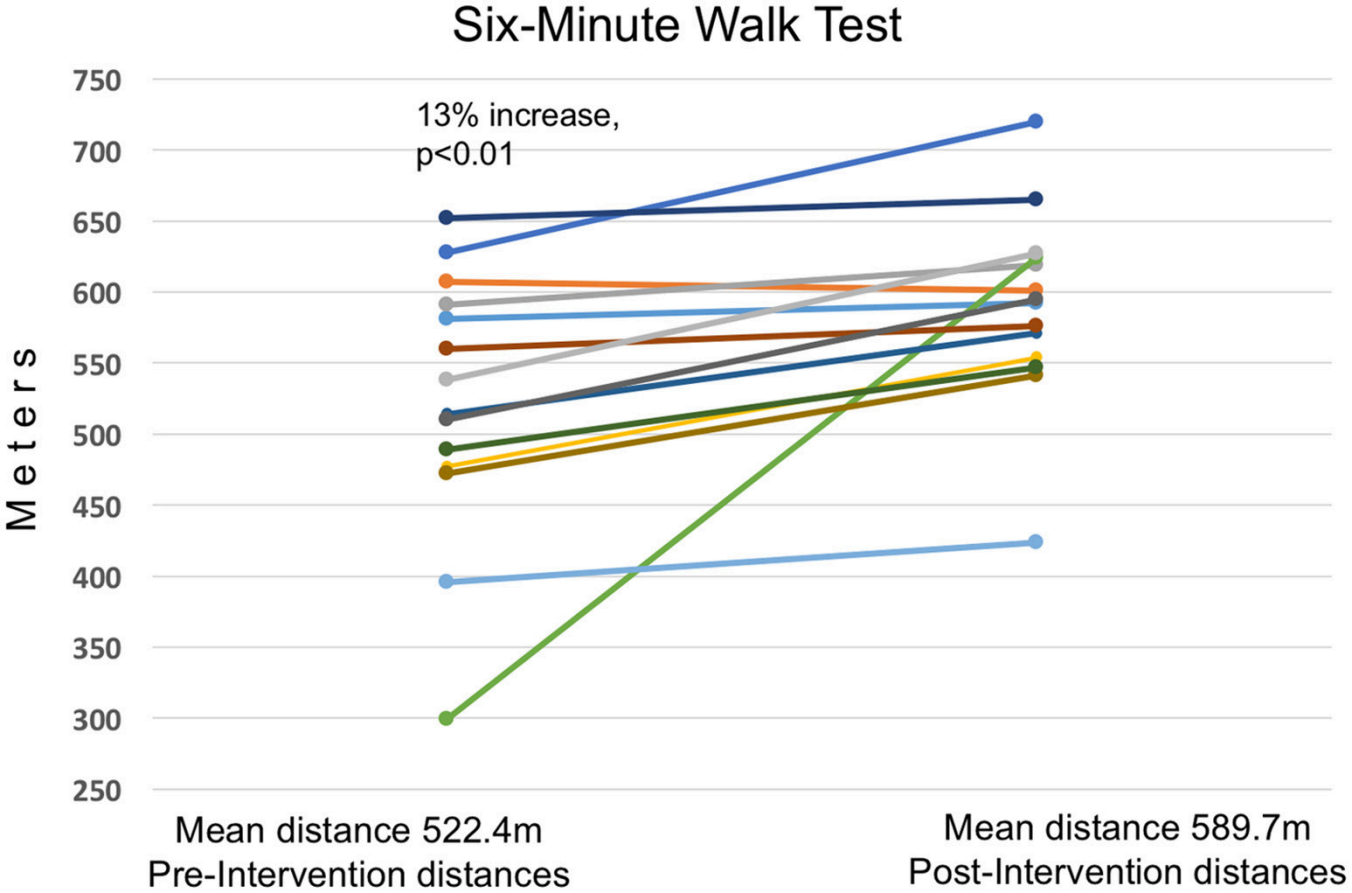
**Thirteen Percent Increase in 6MWT Distance**. Increased distance walked in the 6MWT in meters, from prior to the intervention to after the 4-month Qigong training. Average distance walked increased by 67 m (13%). Significant at a *p* ≤ 0.01 level using Students *t*-test. There was no correlation between improvement in distance walked and pre-intervention scores.

**Figure 5:**
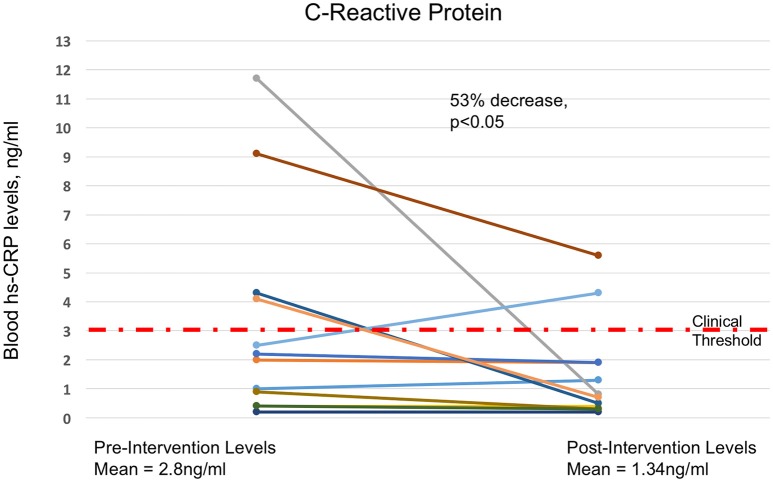
**Fifty-three Percent Drop in Blood hs-CRP Levels**. Decreases in blood levels of hs-CRP, from prior to the intervention to after the 4-month MM training. There was a 53% decrease in average hs-CRP levels (*p* ≤ 0.05 using Students *t*-test), with 10 out of 14 decreasing and 2 remaining at sub-clinical levels. Dashed red line marks the 3 mg/l clinical threshold.

**Figure 6:**
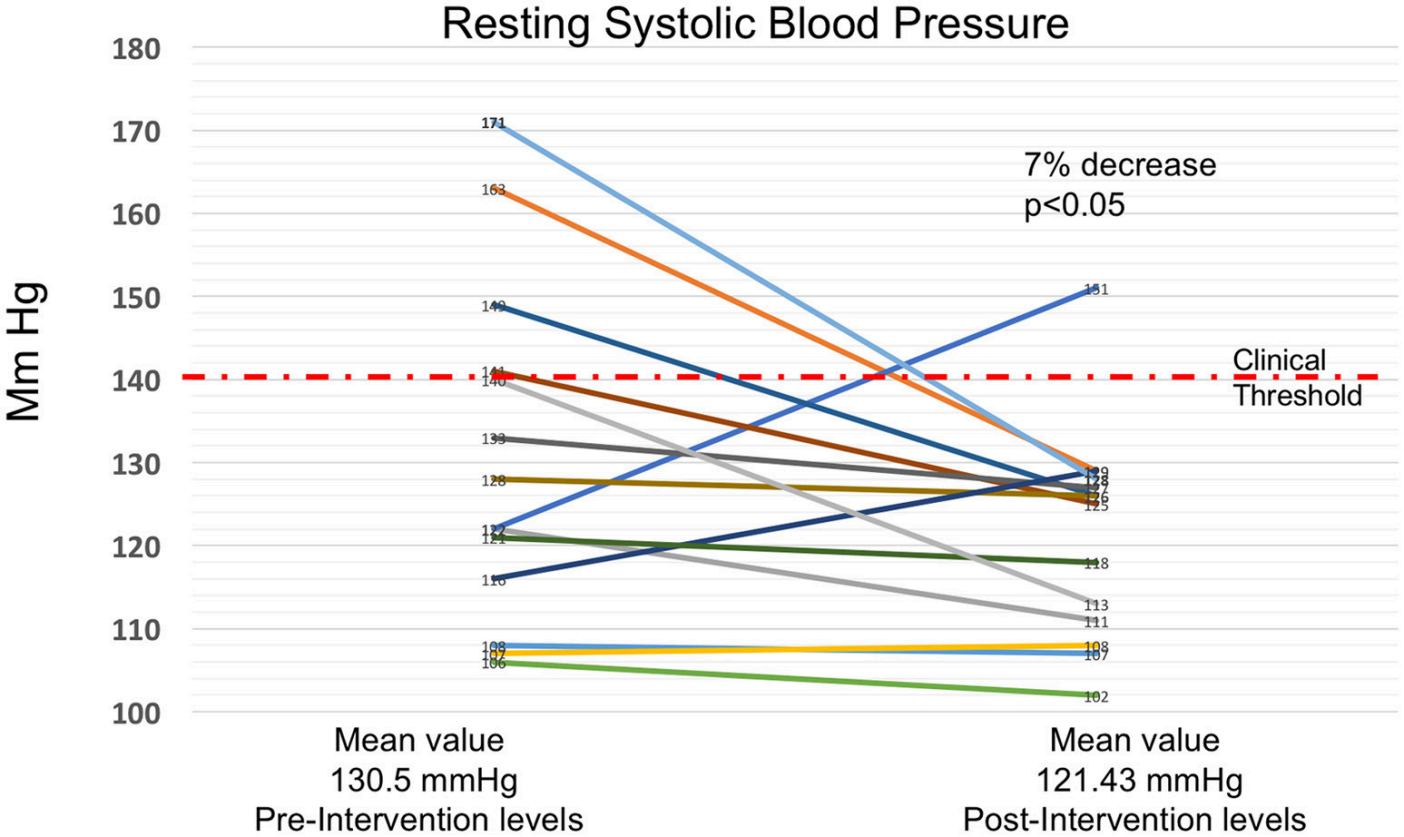
**Seven Percent Decrease in Resting Systolic Blood Pressure**. Decrease in resting systolic blood pressure from prior to the intervention to after the 4-month MM training. There was a 7% decrease after the intervention (*p* ≤ 0.05 using Students *t*-test). 11 out of 14 participants showed reduced systolic blood pressure, and the number of participants above the clinical level declined from 4 to 1. 140 mmHg is regarded as the level above which hypertension occurs, and is indicated by the red dashed line.

Figures 4–6 summarize in graphical form the key results of the study. Changes are shown from testing before and after the Qigong training intervention.

## Discussion

### Summary

Our results indicate improved autonomic function, improved exercise tolerance and cardio-pulmonary function, and reduced systemic inflammation. Autonomic dysfunction, reduced functional ability, and increased systemic inflammation are all co-morbidities of COPD. These results are in line with other studies of the effects of Qigong, specifically on respiratory disease (Ng et al., 2011) as well as on other health factors (Jahnke et al., 2010), supporting the possibility that Qigong may be a useful therapeutic intervention for our target population. Elevated blood pressure, systemic inflammation, autonomic imbalance and reduced functional ability are all strong risk factors for a variety of pathologies, especially in the elderly, suggesting that Qigong practice may have preventive and ameliorative health effects in FA exposed to SHCS and possibly in other populations.

### Primary outcomes

Our intervention was effective in the primary outcomes, the 6MWT and hs-CRP. There was a 13% increase in the distance walked in the 6MWT. This indicates that, despite no significant change in FEV1 or FVC, participants had a substantial and significant increase in functional ability. This outcome was not unique to this study (Chan et al., 2011; Ng et al., 2011). The intervention itself contained no form of aerobic exercise or resistance training; we speculate that the change is due to increased efficiency of body use and movement patterns and a more responsive autonomic nervous system. Other studies have shown increases in strength and bone density due to Qigong practice apparently not due to conventional exercise factors (Lin, 2007; Wayne et al., 2007).

There was a more than 50% decrease in average hs-CRP. 10 of 14 participants improved, 2 stayed the same at low levels, and two increased. All 4 participants with levels over the clinical norm (3.0) reduced substantially, 3 to below the clinical threshold. These results are likely of clinical relevance. In a cohort of women, average age 68, systemic inflammation would be expected to increase with advancing age, and is a risk factor for the development of many diseases of the elderly, including cardiovascular disease, COPD, diabetes, and cancer (Il'yasova et al., 2005; Franceschi et al., 2007; Guarner and Rubio-Ruiz, 2014). Inflammation is associated with autonomic dysfunction (Black, 2003; McGovern and Mazzone, 2014; Mirakaj et al., 2014), so this result is compatible with the hypothesis that the reduction in hs-CRP may be due in part to improved autonomic function. Studies of qigong have shown positive effects on inflammation as well as AD (Lee et al., 2005; Jahnke et al., 2010), and there are both speculative (Pavlov and Tracey, 2012) and well-established (Felten, 1999) links between the autonomic and immune systems which could provide a mechanism for these results.

### Secondary outcomes

Secondary outcome measures provided an opportunity to look at the impact of qigong on conditions co-morbid with COPD. These measures included blood pressure, resting and post-6MWT heart rate, blood oxygen saturation, the COPD Assessment Test (CAT), spirometry, Borg Dyspnea Inventory, the COMPASS 31, the Zung Inventories of Anxiety and Depression, the Multidimensional Assessment of Interoceptive Awareness (MAIA), and Vitamin D.

Cardiovascular measures improved substantially. There was a significant decrease of 7% in average resting systolic blood pressure. 11 out of 14 participants showed reduced systolic blood pressure, and the numbers of subjects above the clinical threshold of 140 mgHg reduced from 4 to 1. There was also an almost significant 12% drop in average pulse pressure. Although the decrease in diastolic pressure did not reach significance, in all three blood pressure-related measures (systolic, diastolic and pulse pressures), the number of participants above accepted clinical range diminished substantially, a result which may be meaningful in clinical terms. Also, resting heart rate decreased significantly by 6%. Since the intervention did not include any forms of conventional physical exercise known to decrease blood pressure or resting heart rate, we speculate that improved autonomic cardiovascular control may be responsible. This hypothesis is supported by the fact that there was a substantial (42%) increase in the change of heart rate after the 6MWT. Although this change did not quite reach significance, it is evidence of a more dynamic cardiovascular autonomic control.

In comparing the graphs of the changes in the 6MWT, hs-CRP and systolic blood pressure, we note that in the measures having a clinical threshold, the change was toward a normal clinical level rather than an across-the-board change. In systolic blood pressure and hs-CRP, values in the normal range showed relatively little change whereas values outside clinical range moved into normal range, with the amount of change approximately proportional to the prior deviation from normal. However, the 6MWT by contrast showed a fairly uniform amount of change regardless of baseline value. Qigong practice claims to normalize physiological variables rather than simply to reduce or increase them in a linear fashion (Cohen, 1999); these results are consistent with this claim.

In addition to objective measures, we included questionnaires to evaluate self-reported autonomic symptoms, affective state and bodily awareness. These are important for evaluating quality of life and changes in cognitive and affective functioning.

The COMPASS 31, a questionnaire evaluating general autonomic function, dropped from 15 to 11.5. This 24% change was significant (*p* ≤ 0.05), and supports the hypothesis of improved autonomic function as a result of the intervention.

The Zung Anxiety score dropped from 29.4 to 26.9 (*p* ≤ 0.05), a significant 8% drop. Since anxiety is associated with autonomic imbalance (increased sympathetic and reduced parasympathetic activity), this result too is compatible with improved autonomic function, as well as being important for better health and quality of life.

The MAIA is designed to measure the degree of awareness a person has of their bodily feelings, and the comfort they have with these feelings. This is hypothesized to relate to the capacity for self-regulation (Farb, 2015; Payne et al., 2015). This capacity includes the ability of the prefrontal cortex, insula and anterior cingulate gyrus to modulate arousal in the limbic system and hypothalamus (Critchley et al., 2003; Craig, 2008). The significant 8% increase in the MAIA score is consistent with the hypothesis that our intervention acted in part by improving autonomic functioning via increased top-down control.

### Possible mechanisms

The mechanisms for the long-term pathological effects of SHCS exposure are only partly understood. Our results suggest an overlooked role for the ANS. Cigarette smoke (CS), whether inhaled directly or second-hand, is known to be a principle cause of COPD and its comorbidities as well as other respiratory system disorders (Moritsugu, 2007). In COPD, inflammation, mucous secretion and plasma extravasation are in excess, and antigen responsiveness is inhibited, all together giving rise to pathology. The pulmonary pathology of COPD is accompanied by systemic abnormalities including inflammation and neuro-humoral activation (Andreas et al., 2005), as well as multiple co-morbidities, including cardiovascular, immune, musculoskeletal and affective disorders (Sin et al., 2006; Fabbri et al., 2008). Although only one of the FA recruited for the present study had COPD as defined by the GOLD standard FEV1/FVC<0.70 (Vestbo et al., 2013b), many of them manifested pulmonary dysfunction (as previously noted by Arjomandi) as well as other symptoms that are recognized as co-morbidities of COPD (Vestbo et al., 2013b). Both core pathology and the co-morbidities of COPD are known to be associated with chronic dysfunction of the autonomic nervous system (ANS) (van Gestel and Steier, 2010). In addition, it is recognized that AD adversely influences the immune system (Czura, 2005), predisposing to inflammation, infections and cancer, all of which are associated with COPD (Libert, 2003; Czura, 2005; Pavlov and Tracey, 2015).

Emerging evidence from human epidemiology and animal testing suggests that the presence of nicotine within the body may alter the susceptibility of the nervous system to long-term dysfunction. CS exposure during stress has been shown to strengthen learned fear responses (Davis et al., 2006; Elias et al., 2010; Kutlu and Gould, 2015). In mouse model studies, long-term fear memory is strengthened. In human epidemiological studies, there appears to be an increased likelihood of developing chronic or post-traumatic stress with consequent autonomic dysfunction (van der Velden et al., 2008). ANS dysfunction negatively affects the immune, cardiovascular, respiratory, and musculoskeletal systems. It seems likely that this may reinforce the pathological pulmonary changes triggered by cigarette smoke through its direct effect on lung tissue. In this model the effects of CS on fear learning and consequent ANS imbalance are an intrinsic part of the etiology of COPD and COPD-related symptoms. This concept is a distinct break from most previous literature, which has documented self-medication through nicotine use in those who already have affective disorders. However, there is evidence that nicotine exposure precedes and exacerbates the effects of exposure to stress or trauma (Elias et al., 2010). Since FA are first responders and are at risk for high-stress exposure, we believe that the hypothesis that systemic co-morbidities of COPD may be exacerbated by cigarette smoke exposure at or close to the time of stressful or traumatic events deserves further consideration.

### Generalizability

This study addressed a specific population, FA exposed to second-hand cigarette smoke. This, and the requirement to attend classes, effectively limited the population to females over 49 with reasonable functional ability. The generalizability of these results to the non-FA population, males, younger people, and those with more severe pathology remains to be established; however given the results of other qigong studies with different populations, it appears likely that similar results would be obtained.

However, in comparing our results with those of other studies of MM, we note that the intervention we developed differs from other MM practices in that it emphasizes integrating the principles of MM practice with daily life, rather than setting aside specific times for practice as is done in most studies (Ng and Tsang, 2009b). We believe there are both advantages and disadvantages to this approach, involving both effectiveness and compliance.

As we have said elsewhere (Payne and Crane-Godreau, 2013), we believe that precise and complete descriptions of qigong interventions are essential in order to enable comparisons between different studies. Details of this intervention are available as supplementary materials to this paper, and a full description and analysis of the exercise selection process, the final choice of exercises, and issues of participant compliance will be published separately. Compliance in particular remains a significant issue, and we plan future studies to explore ways of increasing both compliance and effectiveness of this form of intervention.

### Limitations

The large percentage of participants failing to complete the study is a possible source of bias. This study was not designed to accommodate people with significant levels of affective disturbance. In some cases, affective disturbance only became apparent to us after participants had been consented and had begun to undergo testing. Improved screening is indicated in future similar studies. Life-style was claimed as the reason for non-completion of this study by non-Caucasian participants; we are exploring how to effectively reach a more diverse population.

In this study, we did not use a control group. Since our purpose was to determine which MM exercises are most effective and best tolerated, use of a control group would not have been appropriate. This however diminishes the power of results from the pre-and post-intervention tests as it is not possible determine with certainty whether results are due to attention, seasonal or other factors. However, we are currently proceeding with the RCT stage of the study (Phase II), using an attention/health education control group, which will provide a well-controlled validation of the effects of the selected MM exercises. It is however possible that positive results from the testing in Phase I, followed by negative results from Phase II, might reflect the ineffectiveness of digital delivery rather than the ineffectiveness of the intervention. In this case an RCT testing in-person delivery would be indicated.

The 6MWT is known to be influenced by motivation (Southard and Gallagher, 2013). Every precaution has been taken by the researchers to standardize the administration of the test. Nonetheless, in the Selection stage, subjects may be more highly motivated in the post-test situation, and this could skew results. As mentioned above, the use of a control group in the RCT stage will provide a check on these results.

### Future directions

We are currently conducting Phase II of this study, a randomized controlled trial investigating the effectiveness of digital delivery of these practices. Results from this will indicate whether we need to further refine our method of delivery, or whether we should explore ways of making this material immediately available to FA. In Phase II we are using wearable Holter EKG monitors to analyze heart rate variability. These measures will allow us to test further the hypothesis that the benefits of these practices are due to improved autonomic function, as well as to examine the relative contributions of sympathetic and parasympathetic factors to the reduction of systemic inflammation.

## Replication and supplemental materials

Materials necessary for verification and replication of our results, in addition to supplemental materials, have been deposited at the Harvard Dataverse. A list of titles and doi numbers follows; full citations are provided in the bibliography.

- Replication data for study NCT02612389: Raw data on pre- and post-intervention outcome measure results in Excel format. doi: 10.7910/DVN/3L6APT (Payne and Crane-Godreau, 2016f)
- Replication data for study NCT02612389: Video material to supplement MM instruction. doi: 10.7910/DVN/JDAL38 (Payne and Crane-Godreau, 2016b)
- Replication data for study NCT02612389: PDF files of written material supplementing the individual class instruction in MM”. doi: 10.7910/DVN/CQCTS2 (Payne and Crane-Godreau, 2016c)
- Replication data for study NCT02612389: Audio files used in MM instruction. doi: 10.7910/DVN/68LLAZ (Payne and Crane-Godreau, 2016d)
- Supplemental materials for study NCT02612389: Survey Monkey form used for participants to provide feedback on their experiences from the MM intervention training. doi: 10.7910/DVN/PVUYTD (Payne and Crane-Godreau, 2016e)
- Supplemental materials for study NCT02612389: Personal email from Dr. E. McNeely providing unpublished data on demographics of US (FA). doi: 10.7910/DVN/ASBQCV (McNeely, 2016)
- Supplemental materials for study NCT02612389: Consent form. doi: 10.7910/DVN/KDUJXH (Payne and Crane-Godreau, 2016a)

## Ethics statement

The Committee for the Protection of Human Subjects–Dartmouth College approved the study design. This study was carried out in accordance with the recommendations of The Committee for the Protection of Human Subjects–Dartmouth College, with written informed consent from all subjects. All subjects gave written informed consent in accordance with the Declaration of Helsinki. The protocol was approved by the The Committee for the Protection of Human Subjects–Dartmouth College.

## Author contributions

PP: Funding acquisition, conceptualization, methodology, investigation, original draft, review, and editing, visualization. SF: Supervision, methodology, review and editing. DZ: Resource, review and editing. JL: Formal analysis, methodology, review and editing. MC: Funding acquisition, supervision, project administration, conceptualization, methodology, investigation, review, and editing.

## Funding

We gratefully acknowledge funding support from the Flight Attendant Medical Research Institute (FAMRI) through grants CIA 130052, CIA 130051, and CIA 13050.

### Conflict of interest statement

PP and MC in part receive compensation from teaching and consulting relating to Meditative Movement and MC from consulting on flight attendant health concerns; DZ is financially compensated by the lab that provides the biomarker analysis for the study. SF and JL declare no conflict of interest.

## Figures and Tables

**Table:** 

6MWT	Six minute walk test	A standard test for assessing functional ability, the distance walked in six min
AD	Autonomic dysfunction	
ANS	Autonomic nervous system	Portion of the nervous system controlling those physiological functions outside normal voluntary control.
BP	Blood pressure	
CS	Cigarette smoke	
COPD	Chronic obstructive pulmonary disease	Chronic disease involving obstruction of the small airways and alveolar tissue disruption
CRP	C-reactive protein	Bio-marker of systemic inflammation
DVD	Digital video disc	Standard vehicle for presenting video
FA	Flight attendants	
FAMRI	Flight attendant medical research institute	The foremost organization promoting research into medical problems affecting flight attendants.
FEF	Forced expiratory flow	Spirometric measurement, flow of air during forced exhalation
FEV1	Forced expiratory volume one	A measure of lung function: the maximum volume of air breathed out in a forcible exhalation in one second
FVC	Forced vital capacity	A measure of lung function: the total volume of air breathed out in a complete forcible exhalation
GOLD	Global initiative on Obstructive Lung Disease	A research and information group that is the principal global authority on the nature and treatment of COPD
HR	Heart rate	
MAIA	Multidimensional assessment of interoceptive awareness	A questionnaire designed to evaluate several dimensions of interoceptive awareness
MM	Meditative movement	A newly identified form of exercise characterized by a meditative state of mind, deep relaxation, movement, and attention to the breathing. Qigong and Yoga are the best known examples
PEF	Peak Expiratory Flow	The maximum rate of flow achieved in a forced expiration
PI	Principal investigator	
RCT	Randomized controlled trial	The premier form of research study in which participants are randomly assigned to control or intervention group for the purpose of objective assessment of an intervention
SHCS	Second-hand cigarette smoke	Cigarette smoke inhaled by someone other than the smoker; ambient smoke not inhaled directly through the cigarette

## Footnotes

^1^http://www.catestonline.org/english/indexEN.htm
